# Pneumoperitoneum Secondary to Spontaneously Perforated Pyometra

**DOI:** 10.1155/2017/5213123

**Published:** 2017-03-05

**Authors:** Benjamin A. Raymond, Christopher Esper

**Affiliations:** Department of Surgery, University of Pittsburgh Medical Center, Pittsburgh, PA, USA

## Abstract

Pyometra, by definition, is a collection of purulent fluid within the uterine cavity. Incidence has been estimated to range from 0.1% to 0.5%. Typically, this is linked to postmenopausal women; however, it has been linked to premenopausal women with concordant use of intrauterine devices. Based on our knowledge, there have been less than 50 recorded cases reported in the English literature regarding perforation of pyometra resulting in acute abdomen and fewer than 25 resulting in pneumoperitoneum. We report a patient who was evaluated for diffuse peritonitis caused by perforated pyometra who was successfully treated with surgical intervention.

## 1. Introduction

Pyometra, by definition, is a collection of purulent fluid in the uterine cavity [1]. Incidence has been estimated to range from 0.1% to 0.5%. Typically, this is linked to postmenopausal women; however, it has been linked to premenopausal women with concordant use of intrauterine devices [2, 3]. More than 50% of patients with pyometra are asymptomatic; however, if accompanied by impaired drainage via the cervix it can lead to collection of fluid in the uterine cavity and eventual perforation [4]. Classic presentation is the triad of purulent vaginal discharge, postmenopausal bleeding, and lower abdominal pain. Unfortunately, this classic triad described in literature is only seen in 10% of the patient's affected [5]. Based on our knowledge, there have been less than 50 recorded cases reported in the English literature regarding perforation of pyometra resulting in acute abdomen and fewer than 25 resulting in pneumoperitoneum. We report a patient who was evaluated for diffuse peritonitis and pneumoperitoneum caused by perforated pyometra, successfully treated with surgery.

## 2. Case History

An 80-year-old female presented to our community hospital emergency department with acute onset of bilateral lower quadrant abdominal pain with associated nausea and nonbloody, nonbilious emesis. She denied any subjective fevers or chills at home, and she did not have complaints of shortness of breath, cough, or chest pain. She stated that the abdominal pain started suddenly without warning prior in the evening, and she had never experienced abdominal pain or discomfort similar to this in the past. She denied any significant change in bowel or bladder habits; however, both her and her family at the bedside did state she had been having abnormal vaginal discharge that appeared to be purulent and foul smelling approximately four days prior to emergency department evaluation. Past medical history was significant for hypertension, constipation, and iron deficiency anemia. She did not express any significant surgical history, she denied any tobacco, alcohol, or recreational drug use, and she did not have any significant occupational exposures or chronic family illnesses.

On physical examination, the patient was afebrile with an oral temperature of 36.4 degrees Celsius, blood pressure of 145/71, mild tachycardia with a rate of 104, and respiration rate of 24 with oxygen saturation of 98% without the addition of supplemental oxygen. She appeared to be in mild distress and diaphoretic during the duration of the interrogation and physical examination. On general appearance the patient appeared to be well-kept without obvious sequela of poor hygiene or poor nutritional status. Abdominal examination revealed severe abdominal pain in bilateral lower quadrants to both light and deep palpation with associated involuntary guarding, rebound, and rigidity concerning for gross peritonitis. There was no evidence of anterior abdominal wall hernias or masses appreciated. Rectal exam was negative for occult blood and revealed no palpable lesions or masses. Soft stool was found in the rectal vault. Bimanual exam was performed with the expression of foul smelling, nonbloody purulent fluid from within the vaginal cavity without significant cervical motion or adnexal tenderness. Blood testing revealed no significant lactic acidosis with a measured lactic acid level of 1.1 mmol/L. Complete blood count with differentiation revealed a significant leukocytosis of 20,800 × 10^6^/L with 80% PMNs without bandemia. Complete metabolic panel was significant for an elevated blood urea nitrogen of 46 mmol/L and creatinine 1.23 *μ*mol/L (baseline 1.0). Of note the patient did have a preoperative albumin level of 3.1 g/dL, which is an overall indication of the patient's overall nutritional status. Urinalysis was performed and was void of overt infection.

**Figure 1:**
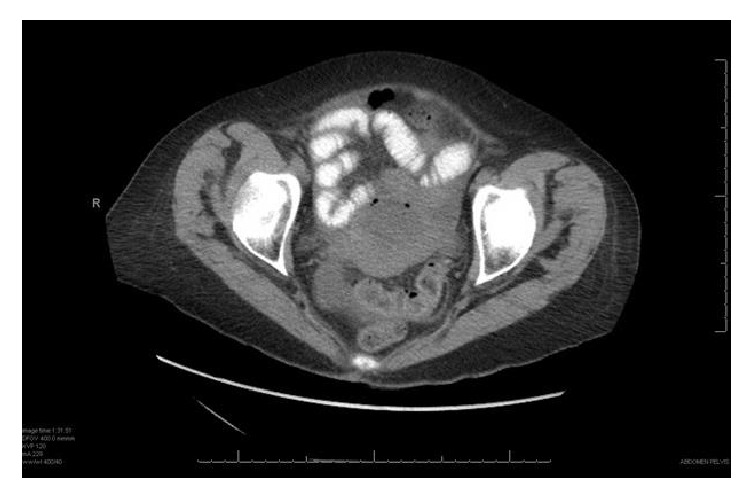
CT abdomen/pelvis showing pneumoperitoneum as well as air within the wall of the uterus.

A CT abdomen and pelvis with both oral and IV contrast (Figure 1) was performed which revealed free intraperitoneal air concerning for perforation. There was also an additional note of foci of air in the mid pelvis questionably within the wall of the uterine fundus. The patient was taken to the OR emergently for exploratory laparotomy, where upon entering the peritoneal cavity there was 600 cc of purulent fluid encountered. This was suctioned and irrigated copiously with normal saline until it was clear; unfortunately, a wound culture was not obtained at this time. The small bowel and large bowel were examined in their entirety without significant pathology noted. The liver, gallbladder, stomach, and spleen were also evaluated and found to be without significant pathology. Thorough examination of the pelvis revealed a hyperemic uterine fundus with associated necrosis and two small areas of perforation for which purulent material was easily identified. These areas were controlled with figure of eight sutures and gynecology was consulted for intraoperative evaluation. At their discretion, they performed a total abdominal hysterectomy with bilateral salpingooophorectomy without complication. Again, the abdomen was evaluated for additional signs of pathology and was copiously irrigated with normal saline and a 10-French flat Jackson Pratt drain was placed in the pelvis. The peritoneum and fascia were closed and a vacuum assisted wound device was utilized secondary to gross contamination of the intraperitoneal cavity and subsequent concern for potential wound infection.

Due to the patient's septic clinical picture, she was placed on broad spectrum antibiotics and antifungals in the form of IV vancomycin, Flagyl, Mefoxin, and Diflucan. These medications were continued for a total of 7 days until leukocytosis resolved and the patient had improvement in her hemodynamic stability. The patient had an uneventful postoperative course and was kept in the intensive care unit for a short period of time before being transferred to the general medical floor and making a full recovery. The patient was discharged home in stable condition on postoperative day #9.

Gross pathological examination of the specimen revealed two perforated areas within the uterine fundus with associated necrosis. The endometrium near the fundus was markedly dilated and hemorrhagic with gross purulence noted. The myometrium at the area of the perforation was vanishing thin; however, away from the area of perforation, the myometrium measured approximately 1 cm in thickness, and the endocervical canal as well as the cervix was without significant pathology. Final pathology of the uterus revealed a perforated pyometra with severe acute and chronic inflammation and transmural necrosis, negative for underlying neoplasia. At this point the consensus was that the most probable cause of pyometra was postmenopausal changes with resulting stenosis of the cervix.

## 3. Discussion

Pyometra is an uncommon clinical condition, which typically occurs in postmenopausal women although it occurs in women of childbearing age as well, and results from the collection of purulent material within the uterine cavity when the natural drainage via the cervix is compromised [6]. Common causes of pyometra included malignancies of the genital tract, which makes up approximately 35% of cases, radiotherapy, and benign conditions such as infection and congenital cervical abnormalities [7]. The accumulation of material in the uterine cavity with compromised natural drainage will lead to the thinning of the uterine walls, which under pressure can cause perforation, pneumoperitoneum, and subsequently an acute abdomen [8]. The most common location for perforation is the uterine fundus, which was the case for this particular patient as well. As with most other case reports of this type, the diagnosis was made intraoperatively; however high clinical suspicion should be warranted with findings of acute abdomen which are present with generalized peritonitis, fever, vomiting, and gynecological symptoms such as vaginal bleeding or purulent vaginal discharge. To date there have been 36 reported cases of spontaneous uterine rupture secondary to pyometra, 35 cases (97%) presented with abdominal pain, 11 cases (31%) presented with fever, and 10 cases (27%) presented with emesis. It has been estimated that the mortality from spontaneous perforation of pyometra is excess of 40%, so expedited supportive care and urgent surgical management including hysterectomy with bilateral salpingooophorectomy are warranted [9]. Unfortunately, in this particular case intraoperative cultures of the purulent material within the abdominal cavity or emanating from the perforated uterus were not obtained, but such results could have identified a causative organism and guided further antibiotic or antifungal treatments.
